# Fragile DNA Motifs Trigger Mutagenesis at Distant Chromosomal Loci in *Saccharomyces cerevisiae*

**DOI:** 10.1371/journal.pgen.1003551

**Published:** 2013-06-13

**Authors:** Natalie Saini, Yu Zhang, Yuri Nishida, Ziwei Sheng, Shilpa Choudhury, Piotr Mieczkowski, Kirill S. Lobachev

**Affiliations:** 1 School of Biology and Institute for Bioengineering and Bioscience, Georgia Institute of Technology, Atlanta, Georgia, United States of America,; 2 Department of Genetics, School of Medicine, Carolina Center for Genome Sciences, Lineberger Comprehensive Cancer Center, University of North Carolina, Chapel Hill, North Carolina, United States of America; National Cancer Institute, United States of America

## Abstract

DNA sequences capable of adopting non-canonical secondary structures have been associated with gross-chromosomal rearrangements in humans and model organisms. Previously, we have shown that long inverted repeats that form hairpin and cruciform structures and triplex-forming GAA/TTC repeats induce the formation of double-strand breaks which trigger genome instability in yeast. In this study, we demonstrate that breakage at both inverted repeats and GAA/TTC repeats is augmented by defects in DNA replication. Increased fragility is associated with increased mutation levels in the reporter genes located as far as 8 kb from both sides of the repeats. The increase in mutations was dependent on the presence of inverted or GAA/TTC repeats and activity of the translesion polymerase Polζ. Mutagenesis induced by inverted repeats also required Sae2 which opens hairpin-capped breaks and initiates end resection. The amount of breakage at the repeats is an important determinant of mutations as a perfect palindromic sequence with inherently increased fragility was also found to elevate mutation rates even in replication-proficient strains. We hypothesize that the underlying mechanism for mutagenesis induced by fragile motifs involves the formation of long single-stranded regions in the broken chromosome, invasion of the undamaged sister chromatid for repair, and faulty DNA synthesis employing Polζ. These data demonstrate that repeat-mediated breaks pose a dual threat to eukaryotic genome integrity by inducing chromosomal aberrations as well as mutations in flanking genes.

## Author Summary

Eukaryotic chromosomes include regions that are susceptible for breakage and rearrangements. Repeats that can adopt non-B form DNA secondary structure are often found to be responsible for the induction of *rearrangements*. Here, we demonstrate that inverted repeats and GAA/TTC breakage sites are also sources of point mutagenesis that can spread to the genes located at long distances from the repeats. Remarkably, repair of the break involving error-prone synthesis restores inverted repeats making them a long-term resource of mutations. These results demonstrate that chromosomal regions with breakage motifs have a high potential for structural rearrangements as well as a tendency to accumulate nucleotide polymorphisms. Increased genetic changes in such regions may alter the rate of changes at the evolutionary scale and contribute to the development of diseases.

## Introduction

Chromosomal instability and mutagenesis are two fundamental processes that alter prokaryotic and eukaryotic genomes. The deleterious consequences of excessive DNA perturbations are hereditary diseases and cancer in humans (reviewed in [1], [2], [3]). At the same time, a fine balance between acquiring genetic changes and restoring original DNA content is paramount for organismal development, adaptation, polymorphism and evolution (for example [4], [5], [6]).

Double-strand breaks (DSBs) in DNA are a driving force for both chromosomal instability and accumulation of mutations. DSBs are a well-established source of a variety of chromosomal aberrations including translocations and copy number variations [7], [8]. It has also become evident from studies in bacteria and yeast that DSB formation and repair are associated with an increased level of mutations, even during homologous recombination which was considered to be an error-free process. In *E.coli*, the role of DSB formation in the induction of mutagenesis was first inferred based on the requirement of RecA and RecBCD for the occurrence of adaptive mutations in the *Lac*Z gene [9] and was later directly demonstrated by using I-*Sce*I endonuclease-induced breaks [10]. In yeast, elevated levels of base substitutions and frame shift mutations were shown to be due to DSB repair in meiosis [11] and as a result of induction of DSBs in mitotically-dividing cells as shown in gene conversion (GC) [12], [13], break-induced replication (BIR) [14] and single-strand annealing (SSA) assays [15]. The proposed mechanism for break-induced mutagenesis, surmised from these studies, involves the formation of long regions of single-stranded DNA (ssDNA) as a result of DSB end resection. Mutations arise during error-prone synthesis either across the damaged ssDNA template or during synthesis following invasion into the undamaged donor strand. There are two lines of evidence supporting this mechanism. First, Yang et al. [15] have shown in yeast, that single-stranded DNA is drastically more prone to the accumulation of mutations with and without treatment with DNA damaging agents than double-stranded DNA. Second, in several studies, mutagenesis was shown to be fully or partially dependent on highly inaccurate translesion polymerases (TLS). The bacterial TLS polymerase, DinB is responsible for 85% of the mutations triggered by DSB repair during adaptive mutagenesis [16]. In yeast, depending on the assay and nature of mutations, DSB-induced mutagenesis is either completely (SSA [15]), partially (GC next to the DSB site and BIR [12], [13], [14]) or not (classical GC assay [17]) attributed to the activity of the error-prone TLS polymerase, Polζ.

Problems encountered by DNA replication machinery are a major source of spontaneous chromosomal breakage in eukaryotes, estimated to be approximately 10 DSBs per cell cycle in human cells (reviewed in [18]). Certain chromosomal regions, the fragile sites, often containing secondary structure-forming repeats, are susceptible to breakage especially under conditions of replication stress [19]. The mutagenic potential of replication-associated breaks has not been studied in detail. It is also unknown what the level of breaks during replication should be for mutagenesis to be manifested. The latter is important considering the fact that in previous studies mutagenesis was detected under conditions of extremely high level of DSBs, reaching up to 100% as seen in the case of site-specific endonucleases. Whether fragile motifs on their own or under conditions of replication stress could be a potent endogenous source of mutations remains to be established.

In this study, we investigate the mutagenic potential of two sequence motifs, inverted repeats and GAA/TTC tracts, which are natural chromosomal fragile sites [20], [21] under conditions of unperturbed and compromised replication. Long inverted repeats can adopt non-B DNA secondary structures such as hairpins and cruciforms owing to their internal symmetry [22]. They are a potent source of genome rearrangements in both prokaryotes and eukaryotes including humans [23]–[26]. We have previously demonstrated that in yeast a 320 bp *Alu*-quasi-palindrome triggers gross chromosomal rearrangements by inducing special type of DSBs that have hairpin-capped termini [21], [26]. The hairpin ends are a substrate for opening and processing by Sae2 and the Mre11/Rad50/Xrs2 (MRX) complex. In *Δmre11*, *Δrad50*, *Δxrs2*, or *Δsae2* mutants, the resection of broken ends is completely blocked, giving rise to inverted dimers. GAA/TTC tracts adopt another kind of non-canonical DNA structure, namely, H-DNA or triplex DNA (reviewed in [27]). The triplex secondary structure is a driving force for the expansions of GAA tracts, a phenomenon responsible for Friedreich's ataxia in humans [28]. Triplex-adopting sequences, including GAA/TTC repeats, are also responsible for breakage and induction of recombination and rearrangements in bacteria, yeast and humans [20], [29]–[34]. Using yeast as an experimental system, we previously demonstrated that triplex structure-imposed replication problems can contribute to breakage at long GAA/TTC tracts [20]. At the same time, GAA-mediated breaks can occur in non-dividing cells where transcription is an important determinant of DSBs [35], [36]. H-DNA forming sequences are mutagenic in yeast and mammalian systems [34], [37]–[39], albeit, direct evidence that repeat-induced fragility is the reason for mutagenesis in the vicinity of the repeats remains to be found.

In this work, we demonstrate that increased break formation at the location of inverted repeats causes mutagenesis at distances up to 8 kb away from the DSB site. The accumulation of mutations requires the Sae2 protein, indicating that resection and generation of long ssDNA is a critical parameter for this phenomenon. We have found that error–prone synthesis involving the translesion polymerase Polζ during repair is primarily responsible for the observed mutagenesis. We also show that in replication-deficient strains the triplex-adopting GAA/TTC repeats are associated with hypermutability at distant loci, suggesting that a similar mechanism of mutagenesis can operate at repeat-associated chromosomal break sites under conditions of replication stress. These data demonstrate that secondary structure-mediated breaks pose a dual threat to eukaryotic genome integrity by inducing chromosomal aberrations and mutations extending to distant chromosomal sites. It is conceivable that the mechanisms of DSB-induced mutagenesis uncovered in this study are also relevant to human evolution, polymorphism and tumorigenesis.

## Results

### Experimental system

**Figure 1:**
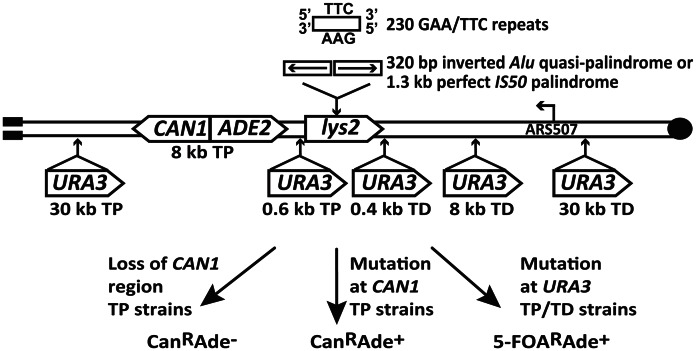
Experimental system to study fragile motif-induced mutagenesis. *Alu*-quasi-palindrome, *IS50-*palindrome or GAA/TTC repeats were inserted into *LYS2* gene on the left arm of chromosome V. Positions of *CAN1* and *URA3* reporters located telomere-proximal (TP) or telomere-distal (TD) to the repeat insertion are shown. The position of the *ARS507* and the direction of replication fork migrating through the repeat region are indicated. Breakage at the location of secondary-structure-adopting repeats can lead to loss of 43 kb telomere-proximal deletion resulting in red-colored Can^R^Ade^−^ clones. Mutations in *CAN1* reporter will yield white-colored Can^R^Ade^+^ isolates. Mutations in *URA3* gene will give rise to colonies resistant to medium containing 5-fluoorotic acid (5-FOA^R^).

The experimental system used to assess the mutagenic potential of fragile inverted and GAA/TTC repeats in this study is based on the GCR assay described in [20], [26]. Briefly, the *LYS2* gene containing the fragile motifs was inserted 43 kb from the telomere on the left arm of chromosome V in haploid yeast strains (Figure 1). There are no essential genes between the left telomere and the *LYS2* gene. *CAN1* is located 8 kb telomere-proximal to the *LYS2* gene. The insertion of *ADE2* between *CAN1* and *LYS2* allows for the differentiation between two types of events on media containing canavanine and low amounts of adenine. Breakage at the location of structure-forming repeats leads to the loss of the terminal 43 kb of the chromosomal arm containing both *CAN1* and *ADE2*, resulting in canavanine-resistant red-colored colonies (Can^R^Ade^−^). On the other hand, mutations in *CAN1* are manifested as white-colored canavanine-resistant colonies (Can^R^Ade^+^) (Figure 1). The correlation between colony color and the requirement of adenine for growth was verified by replica plating the Can^R^ colonies to media lacking adenine. The three fragile motifs inserted into *LYS2* were 100% homologous inverted *Alu* repeats, 320 bp each with a 12 bp spacer (*Alu*-IRs); 100% homologous *IS50* palindromic repeats (*IS50*-PAL) , 1.3 kb each; and 230 repeats of GAA/TTC in the orientation wherein the GAA sequence is the template for the lagging strand synthesis.

To estimate how far mutagenesis can extend from the break site, *URA3* was inserted into chromosome V telomere-proximal (TP) 0.6 kb and 30 kb away from the repeats, and telomere-distal (TD) 0.4 kb, 8 kb and 30 kb away from the repeats (Figure 1). Mutations in *URA3* were measured on 5-fluoroorotic acid-containing media lacking adenine (5-FOA^R^ Ade^+^), allowing us to preferably select these events in contrast to GCRs that give rise to 5-FOA^R^ Ade^−^ colonies.

### A defect in DNA replication leads to increased *Alu*-quasipalindrome-induced breakage and mutagenesis at the *CAN1* locus

Mutation levels in the *CAN1* locus in wild-type strains with inverted *Alu-*quasipalindrome are not different from strains that lack the sequence motif. We wanted to determine whether addition of replication stress will enhance the fragility potential of these repeats and increase mutagenesis. In a screen for mutants that exhibit an increased level of hairpin-capped DSBs we identified the *pol3-P664L* allele that affects the functions of replicative polymerase δ responsible for synthesis of the lagging strand [40]. The *P664L* mutation is located in the polymerase domain of Polδ [41] and the yeast strains carrying this mutant allele exhibit temperature-sensitive growth at 37°C (data not shown). The rate of *CAN1* region loss in strains containing *Alu*-quasipalindrome was 40-fold higher in *pol3-P664L* mutants than in wild-type (Table 1). Moreover, *pol3-P664L* strains with *Alu*-IRs exhibited elevated levels of mutagenesis in *CAN1* loci located 8 kb away from the DSB site. Notably, the mutagenesis was completely dependent on the presence of fragile motifs, suggesting that the mutator phenotype is not a feature of the *pol3* allele but rather is a consequence of increased breakage. It is important to note that in *pol3-P664L* strains without the *Alu*-quasi-palindrome, the relative rate of arm loss was nearly 3-fold higher than in wild-type strains. However, the fragility due to deficiency in Polδ is not high enough to induce mutagenesis.

**Table 1:** Polζ- and Sae2-dependent mutagenesis by *Alu-*quasi-palindrome in replication mutants.

Genetic background	Rate of arm loss×10^−7^ (Can^R^ Ade^−^)		*CAN1* mutation rate×10^−7^ (Can^R^ Ade^+^)	
	No repeats	Inverted repeats	No repeats	Inverted repeats
wild-type	0.05 (0.03–0.06)^a^	520 (450–630)	3 (1–6)	5* (3–7)
*pol3-P664L*	0.14 (0.05–0.2)	21000 (14000–27000)	5 (3–9)	60* (50–80)
TET*-POL3*	0.6 (0.5–0.8)	12000 (10000–14000)	8 (7–13)	140* (90–200)
TET*-RFA2*	20 (11–30)	8600 (6600–15000)	13 (8–23)	92* (35–190)
Δ*rev3*	0.05 (0.03–0.1)	643 (280–2200)	2 (1–4)	2 (1–2)
Δ*sae2*	0.4 (0.3–0.6)	1600 (1100–2200)	2 (1–3)	3 (2–4)
*pol3-P664LΔrev3*	0.3 (0.2–0.5)	24000 (21000–31000)	3 (2–5)	8# (5–10)
*pol3-P664LΔsae2*	8 (5–10)	15000 (11000–21000)	5 (4–8)	7# (6–9)
TET*-POL3Δrev3*	0.7 (0.5–1)	8700 (7000–9600)	8 (7–11)	18# (12–24)
TET*-POL3Δsae2*	4 (2–5)	12500 (12000–15000)	4 (3–6)	6# (5–10)
TET*-RFA2Δrev3*	20 (14–27)	7900 (6000–9000)	21 (17–40)	23# (13–30)
TET*-RFA2Δsae2*	13 (10–15)	14000 (12000–19000)	20 (14–30)	18# (16–26)

a Numbers in parentheses correspond to the 95% confidence interval.

* Depicts mutation rates significantly higher than the wild-type strain (P<0.05).

# Depicts mutation rates in *Δrev3* and *Δsae2* strains significantly lower than corresponding replication-deficient strains (P<0.01).

**Figure 2:**
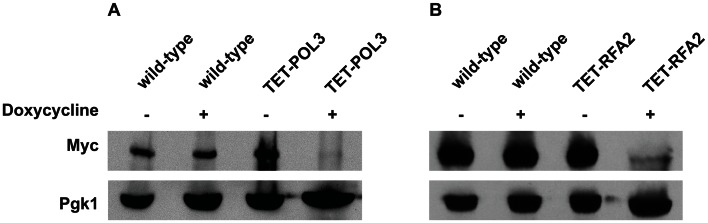
Analysis of protein levels of Pol3 and Rfa2 in the wild-type and tetracycline downregulatable strains. (A) Effect of downregulation of Pol3. (B) Effect of downregulation of Rfa2. Pol3 and Rfa2 were c-Myc tagged at the C-terminus in the wild-type, TET*-POL3* and TET-*RFA2* strains respectively. Proteins were extracted with (+) or without (−) treatment with doxycycline. Pol3 and Rfa2 were detected by Western blot with anti-c-MYC antibody. The protein levels were compared against Pgk1 levels (detected with anti-Pgk1 specific antibody) which acted as the loading control. Upon treatment with doxycycline, Pol3 expression was lowered 10 fold (average of 9, 10 and 12) and Rfa2 expression was lowered 4-fold (average of 4.1, 4.4 and 3.7).

A similar increase in *Alu*-IR-dependent fragility and mutagenesis in *CAN1* gene was observed in strains where the *POL3* expression was under the control of a tetracycline-repressible promoter (*tetO_7_*) [42]. Belli et al., 1998 [42] showed that *tetO_7_*–driven expression of genes in the presence of the antibiotic leads to a reduction in protein levels in comparison to conditions when genes were expressed from their native promoters. Western blotting analysis of c-Myc-tagged Pol3 revealed that upon treatment of cells with doxycycline the protein level was indeed ∼10 fold decreased in comparison with the wild-type level (Figure 2). Hence, we refer to TET-*POL3* as a mutant allele and all further tests were carried out in the presence of doxycycline (see Material and Methods).

We also replaced the native promoter of another replication gene, *RFA2*, that encodes one of the subunits of the single-stranded DNA-binding protein participating in DNA replication and repair, with the *tetO_7_* promoter. Upon downregulation with doxycycline, the expression of Rfa2 was ∼4-fold lower than the wild-type level (Figure 2). Similar to the TET-*POL3* strain, the TET-*RFA2* strain exhibited increased levels of arm loss and mutagenesis (Table 1).

It is important to note that neither the *pol3-P664L* strain nor the TET-*POL3* and TET-*RFA2* strains grown in the presence of doxycycline at chosen concentrations showed sensitivity to DNA damaging agents such as MMS and camptotechin, indicating that they are proficient in DNA repair (Figure S1).

Overall, these data show that mutations at distant loci require the presence of fragile motifs and are dependent on the amount of replication-associated breaks.

### 1.3 kb perfect palindrome induces mutagenesis at the *CAN1* locus even in the wild-type strains

**Figure 3:**
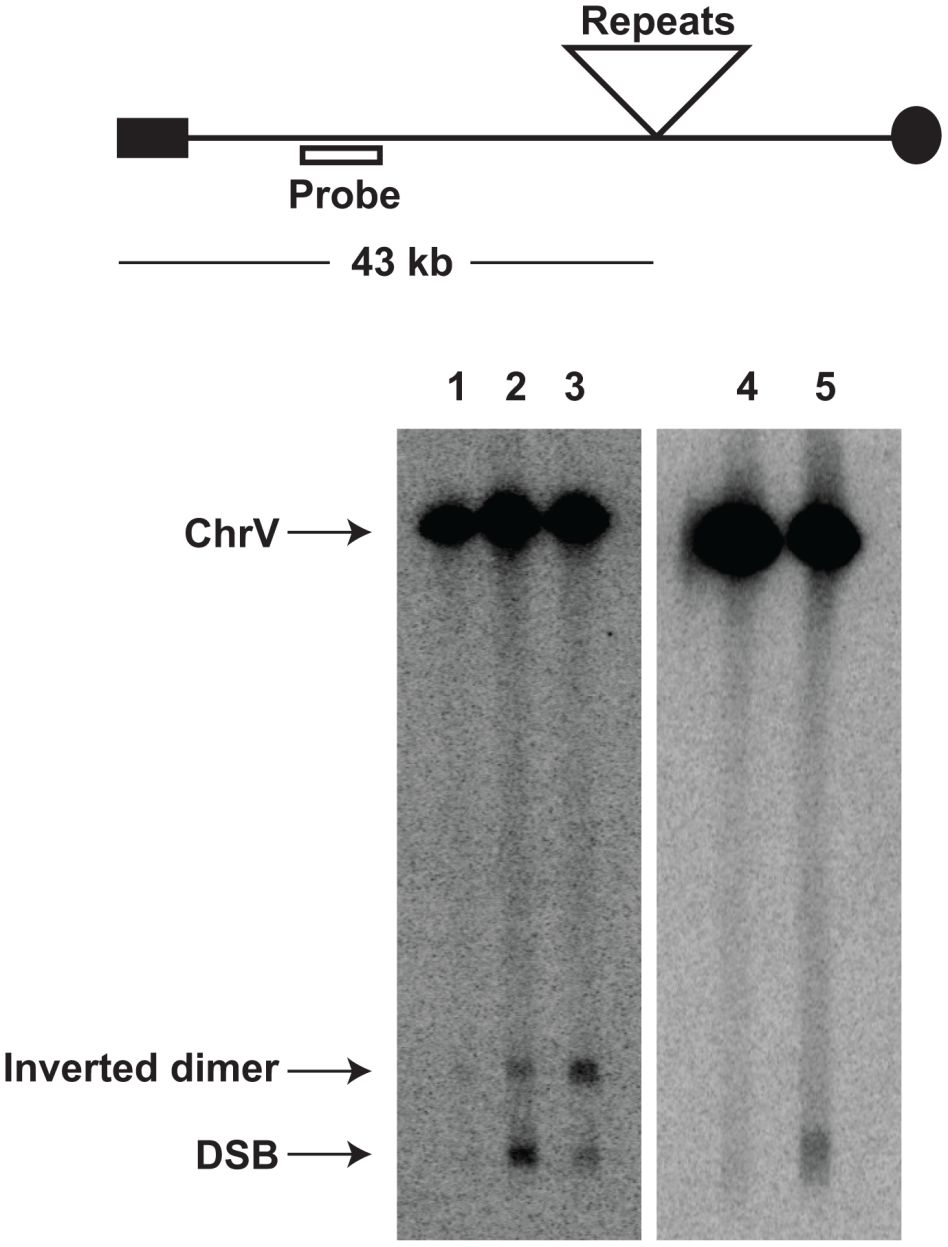
Inverted repeat and GAA/TTC-induced DSB detection in wild-type and mutant strains. Upper panel depicts the relative positions of the inverted repeats and the probe (open rectangle) used. For the detection of inverted repeat-mediated breaks *Δsae2* strains were used as in these mutants the hairpin-capped breaks are not opened and resection is abolished [21]. As a consequence, inverted dimer molecules accumulate in *Δsae2* mutants as previously demonstrated. Contour-clamped homogeneous electric field gel electrophoresis and Southern hybridization were used to highlight the intact chromosome V and the broken fragment. Lanes 1, 2 and 3 depict the *Alu-*IR, *pol3-P664L Alu-*IR, and *IS50-*IR strains respectively. Lanes 4 and 5 depict GAA/TTC_(230)_ and TET-*POL3* GAA/TTC_(230)_ strains respectively. Intact chromosome V, DSB fragments and inverted dimers (in the case of inverted repeats) are indicated.

We addressed directly whether a fragile site can induce mutations at distant loci in replication-proficient strains. This experiment also helps to distinguish which is the key factor in mutagenesis, the level of breakage or repair of broken molecules by faulty replication proteins. The 1.3 kb long *IS50* palindrome was found to induce mutagenesis in *CAN1* when replication was unimpaired (3-fold). The increased length of the interacting arms and the lack of a spacer between them likely create a problem even for intact replication machinery and render this motif highly fragile with a 14-fold increase in GCR rates as compared to the *Alu*-IR strain (Table 2). Consistently, using Southern hybridization, we estimated the level of breakage at this palindrome to be 4.8% (average of 4.6%, 5% and 4.9%) which is ∼3-times higher than in strains carrying the *Alu*-quasi-palindrome (1.6%, average of 1.4%, 1.5% and 1.9%) (Figure 3). Taking into account that a deficiency in Pol3 causes a 7-fold increase in *Alu-*IR-mediated breakage (11%, average of 11%, 10% and 11%) and a 12-fold increase in mutagenesis, it is evident that the levels of DSB formation and not DSB repair by defective replication proteins are the important determinant of mutagenesis.

**Table 2:** Polζ- and Sae2-dependent mutagenesis by *IS50-*perfect palindrome.

Genetic background	Rate of arm loss×10^−7^ (Can^R^ Ade^−^)	*CAN1* mutation rate×10^−7^ (Can^R^ Ade^+^)
wild-type	7000 (6000–9000)^a^	13* (10–19)
Δ*rev3*	9000 (7000–11000)	4# (3–6)
*Δsae2*	6000 (5000–7000)	3# (2–5)

a Numbers in parentheses correspond to the 95% confidence interval.

* Depicts mutation rates significantly higher than the wild-type strain with *Alu-*IR (see Table 1) (P<0.05).

# Depicts mutation rates in *Δrev3* and *Δsae2* strains significantly lower than the wild-type strain (P<0.01).

### Mutagenesis by inverted repeats depends on the distance of the reporter from the DSB site and on the activity of the Sae2 protein

Previously, we have shown that *Alu*-IRs induce DSBs that have hairpin-capped termini [21]. The resection and repair of these DSBs requires the hairpin-opening activity of Sae2 and the Mre11 nuclease [43]. To test if ssDNA generated as a result of 5′-3′DSB end resection is a critical requirement for repeat-induced mutagenesis, the *SAE2* gene was disrupted in *pol3-P664L*, TET*-POL3* and TET*-RFA2 Alu*-IR strains. The level of *CAN1* mutagenesis in *pol3-P664LΔsae2*, TET*-POL3Δsae2* and TET*-RFA2Δsae2* mutants was reduced to levels observed in strains without inverted *Alu*s (Table 1), indicating that mutations are indeed a consequence of DSB resection. Similarly, in the strains carrying *IS50* repeats, mutation rates declined upon deletion of *SAE2* (Table 2).

To determine to what distance the DSB-associated mutagenesis can spread on either side of the fragile site, we inserted the *URA3* reporter 0.4, 8 and 30 kb telomere-distal (TD) and 0.6 and 30 kb telomere-proximal (TP) to *Alu*-IRs in *pol3-P664L* strains (Figure 1). The average length of ssDNA generated via DSB end resection in yeast varies from 2 kb to 10 kb [44]. This predicts that mutations in *URA3* situated past 10 kb should diminish. Consistently, although mutation rates at 0.4 kb, 0.6 kb and 8 kb were approximately the same (10–15-fold higher than in wild-type strain), at 30 kb the rate of *ura3* mutations significantly decreased in TP and TD constructs (Table 3).

**Table 3:** Mutagenesis by fragile *Alu*-IRs depends on the distance of reporter from the DSB site.

		*URA3* Mutation rate×10^−7^ (5-FOA^R^ Ade^+^)					
Location of the reporter from *Alu*-IRs		No repeats		Inverted repeats			
		wild-type	*pol3-P664L*	wild-type	*pol3-P664L*	*Δrev3*	*pol3-P664LΔrev3*
TP^a^ constructs	0.6 kb	1.5 (1–1.9)^c^	1 (0.5–2)	1.3 (0.8–2)	15* (8–20)	0.7 (0.5–0.9)	3# (2–5)
	30 kb	0.6 (0.5–1.3)	1.7 (1.3–2.2)	0.9 (0.6–1.1)	2.9 (2.7–4.7)	ND	ND
TD^b^ constructs	0.4 kb	0.7 (0.4–1.4)	1.1 (0.4–1.9)	1 (0.5–2)	14* (9–17)	1 (0.7–1)	2# (0.9–4)
	8 kb	0.7 (0.6–1)	0.8 (0.7–1.5)	0.6 (0.4–0.8)	9* (6–17)	0.6 (0.3–0.7)	1.4# (0.8–2)
	30 kb	0.6 (0.4–0.9)	1.7 (0.8–3.8)	0.7 (0.2–1.9)	1 (0.8–1.4)	ND	ND

a TP denotes telomere-proximal location of *URA3* with respect to the *Alu*-IRs.

b TD denotes telomere-distal location of *URA3* with respect to the *Alu*-IRs.

c Numbers in parentheses indicate 95% confidence intervals.

* Depicts mutation rates significantly higher than the wild-type strain at the respective loci (P<0.01).

# Depicts mutation rates in *Δrev3* and *Δsae2* strains significantly lower than the *pol3-P664L* strain at the respective loci (P<0.01).

ND - not determined.

The dependence of the efficiency of mutagenesis on the activity of Sae2 and the distance of the reporter from DSB site demonstrates that ssDNA is an intermediate for the occurrence of mutations.

### Increase in mutagenesis observed in replication-deficient and –proficient strains is mostly attributed to the activity of Polζ translesion polymerase

Holbeck and Strathern, [45] and Rattray et al. [12] showed that Polζ translesion synthesis activity is required for the generation of base substitutions in a reporter located 0.3 kb from the site of an HO-endonuclease-induced break. To assess if mutagenesis induced by fragile motifs depends on translesion synthesis, we disrupted the *REV3* gene encoding the catalytic subunit of Polζ [46] in wild-type, *pol3-P664L*, TET-*POL3* and TET*-RFA2* strains with *Alu-IRs* (Table 1). *REV3* disruption in replication-defective strains brought the mutation level in the *CAN1* reporter to almost the level observed in the wild-type strain. In replication-proficient strains carrying *Δrev3*, only a modest 2-fold decrease in *CAN1* mutation rate was observed. Augmented mutation rates in the strains with *IS50* repeats were also dependent on the activity of Polζ (Table 2).

To gain further insight into the spectrum of mutations generated at distant loci as a result of DSB formation by inverted repeats, we sequenced 22–31 independent Can^R^Ade^+^ isolates from wild-type, *pol3-P664L* and *pol3-P664LΔrev3* strains, respectively. In the wild-type strain with *Alu*-IRs, 85% of the mutations were base substitutions and 15% were single base deletions (Table 4 and Table S1.). A similar mutation spectrum was also observed in other studies [47], [48]. This correlates with the lack of increase of *CAN1* mutagenesis in the wild-type *Alu*-IR strain (Table 1), indicating that the observed mutations in replication-proficient strains were a result of spontaneous mutagenesis rather than secondary structure-induced DSBs. In the *pol3-P664L* strain the mutation spectrum was changed. There was a significant increase in the frequencies of base substitutions, particularly G∶C→T∶A and G∶C→C∶G transversions characteristic of Polζ errors during spontaneous mutagenesis [49] (Table 4 and Table S2). Increases in deletions ranging from 1 to 5 bp and complex mutations (two or more changes in a run of 10 bp) were also observed. These types of changes were also previously attributed to the TLS activity of Polζ [48], [50]. A similar mutation spectrum was also seen for Can^R^Ade^+^ clones from strains containing the *IS50*-perfect palindrome (Table 4 and Table S5). Since mutagenesis observed in these strains requires the activity of Polζ, it is likely that error-prone synthesis by the TLS polymerase during DSB repair causes base substitutions as well as deletions and complex mutations. Consistently, errors that could be assigned to the activity of Polζ were suppressed in *pol3-P664LΔrev3* strains (Table 4 and Table S3). We also uncovered large deletions (up to 39 bp) and a duplication of 27 bp flanked by short direct repeats in *pol3* mutants with or without *Alu*-IRs. This is most probably attributed to the defective Polδ. Notably, *pol3-P664L* strains that lack fragile motifs also exhibited complex mutations (Table 4 and Table S4). Taking into account that fragility in *pol3-P664L* without *Alu*-IRs is low, it can be inferred that these changes reflect mutations arising during DNA replication carried out by a faulty DNA polymerase (a process that also might require TLS polymerases [51]) rather than a consequence of error-prone synthesis during DSB repair.

**Table 4:** Mutation spectra in *CAN1* reporter.

	Mutation rate×10^−7^				
Class of mutation^a^	wild-type+*Alu*-IR	*pol3-P664L*+*Alu*-IR	*pol3-P664LΔrev3*+*Alu*-IR	*pol3-P664L*−*Alu*-IR	wild-type+*IS50*-PAL
Base substitutions	4.2 (23^b^, 85%^c^)	27 (14, 45%)	3.2 (9, 41%)	2.4 (14, 48%)	9.4(18, 72%)
**- G∶C→T∶A, G∶C→C∶G transversions**	2.0 (11, 41%)	19* (10, 32%)	1.8 (5, 22%)	1.0 (6, 20%)	4.7* (9, 36%)
- other substitutions	2.2 (12, 44%)	8 (4, 13%)	1.5 (4, 18%)	1.3 (8, 27%)	4.7 (9, 36%)
**Frameshifts** d	0.7 (4, 15%)	17* (9, 29%)	0.4 (1, 5%)	0.7 (4, 14%)	2.1* (4, 16%)
**Complex mutations** e	<0.1^g^ (0, 0%)	12* (6, 19%)	<0.4 (0, 0%)	0.5 (3, 10%)	1.6* (3, 12%)
Slippage^f^	<0.1 (0, 0%)	4 (2, 6%)	4.3 (12, 54%)	1.2 (7, 24%)	<0.5 (0, 0%)
Total	5 (27, 100%)	60 (31, 100%)	8 (22, 100%)	5 (29, 100%)	13 (25, 100%)

a Classes of mutations characteristic for Polζ are indicated in bold.

b Parentheses indicate the number of sequenced isolates of each subclass.

c Relative fraction of the isolates of each subclass among the total isolates sequenced.

d Frameshift mutations are insertions and deletions of 1–5 nucleotides.

e Complex mutations are more than one nucleotide change in a span of 10 nucleotides.

f Slippage subclass includes deletions from −15 to −39 nucleotides and duplication of 27 nucleotides flanked by short direct repeats.

g <value reflects less than measurable rate of mutations.

* indicates the classes demonstrating increased rates of mutagenesis due to the activity of Polζ.

Overall, analysis of mutation spectra in wild-type and replication-deficient strains is in agreement with genetic analysis and supports the conclusion that repeat-mediated mutations are generated by error-prone Polζ and do not occur due to faulty synthesis by replicative polymerases.

### GAA/TTC fragile motif also induces mutagenesis at distant chromosomal loci that is partly Polζ dependent

To determine if DSB-induced mutagenesis can be observed at another fragile motif, we assessed *CAN1* mutation rate in strains carrying 230 repeats of the triplex-adopting GAA/TTC (Figure 1). Although the rate of Can^R^ mutations was unaltered in wild-type strains, a 4-fold increase in mutagenesis was detected in *pol3-P664L* and TET-*POL3* strains (Table 5). The level of DSB formation at GAA/TTC repeats in the TET-*POL3* strain was estimated to be 3.3% (average of 3.1%, 3.3% and 3.6%, Figure 3). This is a minimal estimation of GAA/TTC-mediated DSBs since, unlike the situation with palindromic sequences, resection of the broken fragments cannot be prevented by *SAE2* disruption and a proportion of degraded DSBs are excluded from detection.

**Table 5:** Mutagenesis in *CAN1* reporter stimulated by GAA/TTC repeats.

Genetic background	Rate of arm loss×10^−7^ (Can^R^ Ade^−^)		*CAN1* mutation rate×10^−7^ (Can^R^ Ade^+^)	
	No repeats	GAA/TTC_(230)_	No repeats	GAA/TTC_(230)_
wild-type	0.03 (0.01–0.04)^a^	20 (10–30)	3 (3–4)	5 (3–9)
*pol3-P664L*	0.2 (0.1–0.3)	240 (190–260)	5 (3–6)	19* (15–24)
TET*-POL3*	0.6 (0.5–0.8)	180 (130–240)	9 (8–10)	30* (20–50)
Δ*rev3*	0.08 (0.05–0.1)	20 (10–30)	2 (1–2)	3 (2–4)
*pol3-P664L Δrev3*	0.4 (0.3–0.6)	214 (176–266)	4 (3–6)	12# (10–16)
TET*-POL3 Δrev3*	0.7 (0.6–0.9)	160 (140–200)	9 (7–10)	14# (9–16)

a Numbers in parentheses correspond to the 95% confidence interval.

* Depicts mutation rates significantly higher than the wild-type strain (P<0.05).

# Depicts mutation rates in *Δrev3* and *Δsae2* strains significantly lower than corresponding replication-deficient strains (P<0.05).

Similar to *Alu*-IR-mediated mutagenesis, Polζ plays a role in the induction of mutations by GAA/TTC repeats. There was a mild but statistically significant reduction (2-fold) in mutagenesis in *pol3-P664LΔrev3* versus *pol3-P664L* (p<0.05) and TET-*POL3Δrev3* versus TET-*POL3* (p<0.05) strains as determined using an unpaired t-test. Although it is difficult to evaluate the contribution of resection and long ssDNA to GAA/TTC-associated mutagenesis, the involvement of *REV3* suggests that the mechanism underlying mutagenesis in the case of inverted repeats and GAA/TTC fragile sites can be similar.

## Discussion

The induction of DSBs using site-specific endonucleases has been shown to drive mutagenesis [12]–[15]. This study demonstrates that natural chromosomal fragile sites comprising of sequence motifs that can adopt non-B DNA structures are also mutagenic. Under condition of replication stress, the mutagenesis can reach up to the levels caused by deficiency in the mismatch repair system [52]. We also show that the mutations are a consequence of error-prone repair of repeat-induced DSBs. Overall, we establish secondary structure-forming motifs as a potent source of endogenous mutagenesis and reveal the mechanism underlying this phenomenon.

In this study we found that when replication is compromised, *Alu*-quasi-palindrome promotes chromosomal fragility and mutagenesis at *CAN1* and *URA3* reporters located 8 kb from the break site. Mutations were also increased in strains with a perfect *IS50*-palindrome with inherently higher fragility even in replication-proficient strains. We have previously shown that inverted repeats induce hairpin-capped DSBs in replication-proficient strains [21]. We have found that in replication-defective mutants the DSBs mediated by the *Alu-*quasi-palindrome also have hairpin-capped termini (Y. Zhang, N. Saini, Z. Sheng, K.S. Lobachev, in preparation). The opening of the hairpins necessitates the nuclease activity of the MRX complex and Sae2. The requirement of Sae2 for mutagenesis at distant loci unequivocally demonstrates that mutations are a consequence of DSB formation (Table 1 and Table 2). Moreover, these data also implicate the formation of long ssDNA upon resection of DSB ends as the second step in repeat-mediated mutagenesis. ssDNA has been shown to be prone to accumulation of mutations during SSA or in a situation where the telomeres become uncapped [15]. Therefore, it is possible that hairpin-processing generates damaged ssDNA that can serve as a faulty template for synthesis during SSA or GC. Alternatively, the undamaged ssDNA can be involved in strand invasion and mutations could arise due to error-prone synthesis during homologous recombination as suggested in other studies [9], [13], [14]. Error-prone synthesis of the undamaged DNA template in replication deficient strains by Polζ was observed by Northam et al. [51].

**Figure 4:**
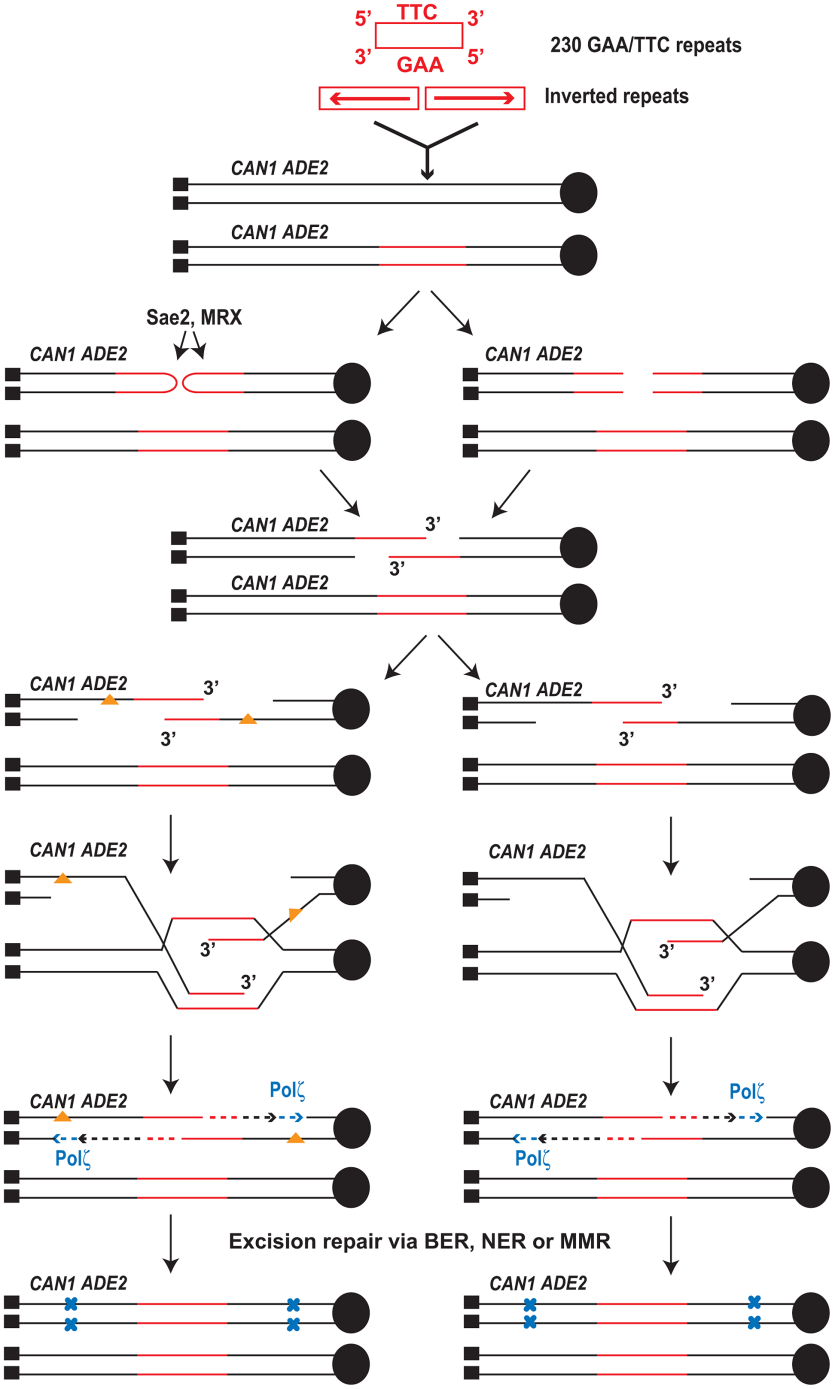
Model for mutagenesis induced by *Alu*-IRs and GAA/TTC repeats. The inverted repeats and 230 repeats of GAA/TTC inserted into *LYS2* are shown in red (not drawn to scale). Centromere (filled black circle) and telomeres (filled black squares) are also shown. Inverted repeats and GAA/TTC repeats trigger DSBs in late S or in G2 wherein the intact sister chromatid is present. The inverted repeats-induced hairpin-capped DSB are processed by Sae2 and the MRX complex (shown on the left). GAA/TTC tracts induce DSBs that have exposed 5′ and 3′ termini (shown on the right). Two scenarios for the accumulation of mutations are shown. On the left, ssDNA generated as a result of extensive resection can accumulate damages (orange triangles). Error-prone synthesis during the fill in reaction will lead to mutations (shown as blue x). On the right, errors can be made by Polζ during synthesis across the undamaged template. Mutations will be incorporated either due to the action of mismatch repair or in next round of DNA replication (not shown).

Although we cannot determine whether mutagenesis is due to accumulation of damage in resected DNA or error-prone synthesis on undamaged template, our data point towards synthesis-dependent strand annealing (SDSA) as the underlying mechanism for mutagenesis (Figure 4). None of the analyzed Can^R^ clones contained interstitial deletions and all of the clones retained intact *Alu*-IRs or *IS50*-palindrome (data not shown). This suggests that SSA is unlikely to operate during *Alu-*IR-mediated mutagenesis and alludes to a template-dependent repair process that involves the undamaged sister chromatid. Thus, we favor a scenario wherein hairpin-capped DSBs are induced in late S or G2 stage of the cell cycle. Upon hairpin opening by Sae2 and MRX, the 3′ end of the resected DSB invades the intact sister chromatid template. The requirement for invasion in mutagenesis is a likely step but ultimately cannot be proven by using *rad51* or *rad52* mutants for two reasons: these strains exhibit a mutator phenotype on their own [49] and Rad51 and Rad52 proteins are required for DSB formation at the *Alu*-IRs in replication-defective strains (Y. Zhang, N. Saini, Z. Sheng, K.S. Lobachev, in preparation). It is conceivable that the invasion event can proceed either as a BIR or as an SDSA event. SDSA is the most probable mechanism owing to the fact that mutations were observed in both TP and TD reporters and that reduced mutation rates were measured at reporters 30 kb from the break site (Table 3). It is important to note that SDSA preserves the original inverted repeats that can trigger additional rounds of breakage and associated mutagenesis. If extrapolated to humans, these observations identify secondary structure-forming repeats as a potent source of mutagenesis that can change the expression of flanking genes during the lifetime of healthy individuals even in the absence of exogenous damage.

Mutations generated in the reporter 8 kb away from DSB site were also strongly dependent on the activity of Polζ (Table 1 and Table 2) indicating that the error-prone translesion synthesis operates during SDSA. This is consistent with the Hirano and Sugimoto, 2006 study that showed that Mec1 kinase is needed to recruit the Polζ-Rev1 complex to the DSB site [53] and other studies where DSB-induced mutagenesis required Polζ [12]–[15]. Analysis of mutations in the *CAN1* locus of the hyper-fragile strains revealed an increase in G∶C→T∶A and G∶C→C∶G transversions, frameshift and complex mutations that are signatures of Polζ (Table 4) [48]–[50].

In this study we also show that DSB-triggering long GAA/TTC repeats induce mutagenesis at distant loci, indicating that a similar underlying mechanism of mutagenesis described above for inverted repeats can operate for triplex-forming motifs. The requirement of Rev3 for mutagenesis is more evident for inverted repeats than for GAA/TTC repeats. It would be interesting to see if other TLS polymerases, Rev1 and Polη, besides Polζ operate in GAA/TTC-associated mutagenesis and to determine if the mutation spectra in GAA/TTC- and inverted repeat-containing strains differ. It is also important to note that in our experimental system, we observe mutagenesis by GAA/TTC tracts only under conditions of compromised replication wherein the repeat-mediated fragility is further increased. In other studies, mutagenesis is induced by GAA/TTC repeats in replication-proficient strains [37], [38]. These discrepancies might reflect the distance of the used reporter from the fragile motif. It is possible that at closer distances, SSA might be the predominant pathway for mutagenesis where mutations introduced by Polζ can be scored above the spontaneous level of mutagenesis, while SDSA requires higher frequencies of breakage and longer ssDNA. This can be checked experimentally in future studies. Our data are also in agreement with the recent study by Shah et al., 2012 wherein GAA/TTC-induced mutations in a closely juxtaposed reporter in Polδ mutants were dependent on Polζ [39].

Overall, this study demonstrates that fragile sequence motifs that are found in eukaryotic genomes, including humans, can be potent inducers of mutagenesis. Thus, secondary structure-adopting repeats can represent a dual threat to DNA stability by changing the structural organization of the genome and causing mutations. Recent studies linking the occurrence of mutations near chromosomal rearrangement break-points in primates and humans suggest that error-prone repair of DSBs can operate during speciation, evolution and tumorigenesis [54]–[57]. Thus it is likely that fragile and mutagenic non B-DNA-forming motifs are contributing factors to these processes.

## Materials and Methods

### Yeast strains

The yeast strains used for the analysis of the inverted repeat-induced mutagenesis were derivatives of the KT19 strain (MATa, *bar1-Δ, his7-2, trp1-Δ, ura3-Δ, leu2-3,112, ade2-Δ, lys2-Δ, cup1-Δ, yhro54c-Δ, cup2-Δ*, *V34205*::*ADE2lys2*::*Alu-IRs*, *V29616*::*CUP1*). GAA/TTC-mediated mutagenesis was measured in strains that were derivative of YKL36 (MATa, *bar1-Δ, his3-Δ, trp1-Δ, ura3-Δ, leu2-Δ, ade2-Δ, lys2-Δ, V34205*::*ADE2lys2*::(*GAA)_230_). Alu-*IRs and GAA repeats were inserted into the *Bam*HI site and the *IS50* palindrome was inserted into *Hpa*I site in *LYS2*. The strains without repeats had an intact *LYS2* gene. For measuring the distance dependence of repeat-induced mutagenesis, *URA3* was amplified from pRS306 with flanking regions for the points of insertion into chromosome V. *URA3* was inserted close to the repeats in *lys2* (586 bp TP and 352 bp TD), ∼8 kb TD of the repeat locus between SGD coordinates 42096 and 42097 and ∼30 kb TP between SGD coordinates 11910 and 11911 and TD between coordinates 64686 and 64687 (Figure 1, Table S6). The *pol3-P664L* allele was created via site-directed mutagenesis using p170 [58]. The mutation *P664L* results in the appearance of the *Ase*I site. The plasmid was digested with *Hpa*I and the mutation was obtained using pop-in pop-out methodology. The mutant shows mild temperature sensitivity at 37°C. The tetracycline promoter construct was obtained from Euroscarf (pCM225). PCR was performed with primers carrying overhangs for *RFA2* and *POL3* promoter regions and one-step integration was used to replace the promoters for *RFA2* and *POL3* (Table S6). *REV3* was replaced with the *kanMX* cassette in wild-type and *pol3-P664L* strain and with the *hph*MX cassette amplified from pAG32 in the TET-*POL3* and TET*-RFA2* strains [59]. *SAE2* was disrupted with the *kanMX* cassette in the wild-type strains and with *TRP1* in *pol3-P664L*, TET*-POL3* and TET*-RFA2* strains.

### GCR and mutation rates estimations

Fluctuation tests were carried out to estimate mutation and GCR rates. The strains were allowed to grow on YPD agar for 3 days at 30°C. The TET*-POL3* and TET*-RFA2* strains were grown on YPD containing 2 µg/ml and 0.1 µg/ml doxycycline, respectively. At these chosen concentrations of doxycycline the proteins are downregulated leading to an increase in fragility without significantly affecting viability of the strains. 14 individual colonies were diluted in 0.25 ml water each and serial dilutions were made to approximately 1∶10000. The cultures were plated on YPD and on L-canavanine (60 mg/L) low adenine (5 mg/L) containing synthetic media in order to obtain approximately several hundred colonies per plate after incubating for 3 days at 30°C. White colonies on canavanine-containing media are indicative of mutations in *CAN1* while red colonies depict GCR events. For mutation rate estimation at *URA3*, the cultures were appropriately diluted and plated on 5-FOA (1 g/L) containing synthetic media lacking adenine. Mutation rates and 95% confidence intervals were calculated as previously described [21].

### DSB detection and quantification

Yeast cells were embedded into agarose plugs at a concentration of 2×10^9^ cells/ml for detection of inverted-repeat-mediated DSBs and at a concentration of 8×10^9^ cells/ml for detection of GAA/TTC-induced DSBs. The chromosomes were separated using contour-clamped homogeneous electric field gel electrophoresis as described previously [36] and transferred onto a nylon membrane. Southern hybridization was carried out using a probe specific to *HPA3* that is telomere-proximal to the repeats. Densitometry analysis was performed using ImageJ (NIH) and the intensity of the broken product was normalized against the intact chromosome V.

### Sequence analysis of mutations at *CAN1*

*CAN1* mutants were obtained by plating approximately 30 individual colonies from two independent isolates for each strain on L-canavanine low adenine containing synthetic media. The Can^R^Ade^+^ isolates were then streaked out on YPD to obtain single colonies from which DNA was extracted. PCR was carried out using primers 60 bp upstream and 158 bp downstream of *CAN1*. The PCR product was sequenced using 4 internal primers for *CAN1* such that the entire gene would be covered at least twice during sequencing. The primers used for sequencing *CAN1* are

can1-o1: 5′CATCTACTGGTGGTGACAAAG3′;

can1-s1: 5′GCCACGGTATTTCAAAGCTTGC3′;

can1-s2: 5′GGCTCTTGGAACGGATTTTC3′;

can1-s3: 5′TGTAGCCATTTCACCCAAGG3′. The sequencing results are depicted in Table 4 and Tables S1, S2, S3, S4, S5.

### Estimation of Pol3 and Rfa2 expression

To quantify the changes in protein expression *POL3* was tagged with 13 copies of c-Myc-epitope tag and *RFA2* was tagged with 9 copies of the c-Myc-epitope tag at the C-terminus in both wild-type and tetracycline downregulatable strains. TET-*POL3* was grown in the presence of 2 µg/ml doxycycline overnight, while TET-*RFA2* was grown with 0.1 µg/ml doxycycline overnight. Total protein was extracted as previously described [60] and separated using SDS-polyacrylamide gel electrophoresis on an 8% gel. After electrophoresis the gel was blotted on a nitrocellulose membrane and probed with an antibody specific to c-Myc (Genscript) and an antibody for Pgk1 (Invitrogen). The membrane was further treated with anti-mouse secondary antibody (Genscript) and chemiluminescent detection was carried out using the protocol described by GE Healthcare. Densitometry analysis was performed using ImageJ (NIH) and the intensity of Pol3 and Rfa2 were normalized against the loading control Pgk1.

## Supporting Information

Figure S1 Sensitivities of the replication-deficient strains to DNA-damaging agents. Four-fold serial dilutions of wild-type strains and the mutant alleles were plated on YPD and YPD containing 1.5 mM MMS and 10 µg/ml camptothecin (CPT). Δ*rad51* was used as a control since it exhibits extreme sensitivity to the drugs used. The middle and bottom panels depict strains grown in the presence of 2 µg/ml and 0.1 µg/ml doxycycline, respectively.
(TIF)

Table S1 Sequences of mutations analyzed in *CAN1* in wild-type strain containing inverted repeats.^ a^ Coordinates of the first nucleotide in the mutated sequence are indicated based on the *CAN1* coding strand sequence. ^b^ sub - base substitutions, indel - insertions or deletions
(DOC)

Table S2 Sequences of mutations analyzed in *CAN1* in *pol3-P664L* mutant strain carrying inverted repeats.^ a^ Coordinates of the first nucleotide in the mutated sequence are indicated based on the *CAN1* coding strand sequence. ^b^ sub - base substitutions, indel - insertions or deletions, complex - complex mutations, slippage- slippage events between short direct repeats that are indicated by underlined sequences.
(DOC)

Table S3 Sequences of mutations analyzed in *CAN1* in *pol3-P664L Δrev3* mutant strain carrying inverted repeats.^ a^ Coordinates of the first nucleotide in the mutated sequence are indicated based on the *CAN1* coding strand sequence. ^b^ sub - base substitutions, indel - insertions or deletions, complex - complex mutations, slippage- slippage events between short direct repeats that are indicated by underlined sequences.
(DOC)

Table S4 Sequences of mutations analyzed in *CAN1* in *pol3-P664L* mutant strain carrying no inverted repeats.^ a^ Coordinates of the first nucleotide in the mutated sequence are indicated based on the *CAN1* coding strand sequence. ^b^ sub - base substitutions, indel - insertions or deletions, complex - complex mutations, slippage- slippage events between short direct repeats that are indicated by underlined sequences.
(DOC)

Table S5 Sequences of mutations analyzed in *CAN1* in wild-type strain carrying *IS50*-perfect palindrome.^ a^ Coordinates of the first nucleotide in the mutated sequence are indicated based on the *CAN1* coding strand sequence. ^b^ sub - base substitutions, indel - insertions or deletions, complex - complex mutations.
(DOC)

Table S6 Sequences of the primers used in this study.
(DOC)
