# Supplementary figures and images for: Fragile DNA Motifs Trigger Mutagenesis at Distant Chromosomal Loci in Saccharomyces cerevisiae
Source: PLoS Genet. 2013 Jun 13;9(6):e1003551. doi: 10.1371/journal.pgen.1003551 (PMC3681665; doi:10.1371/journal.pgen.1003551)

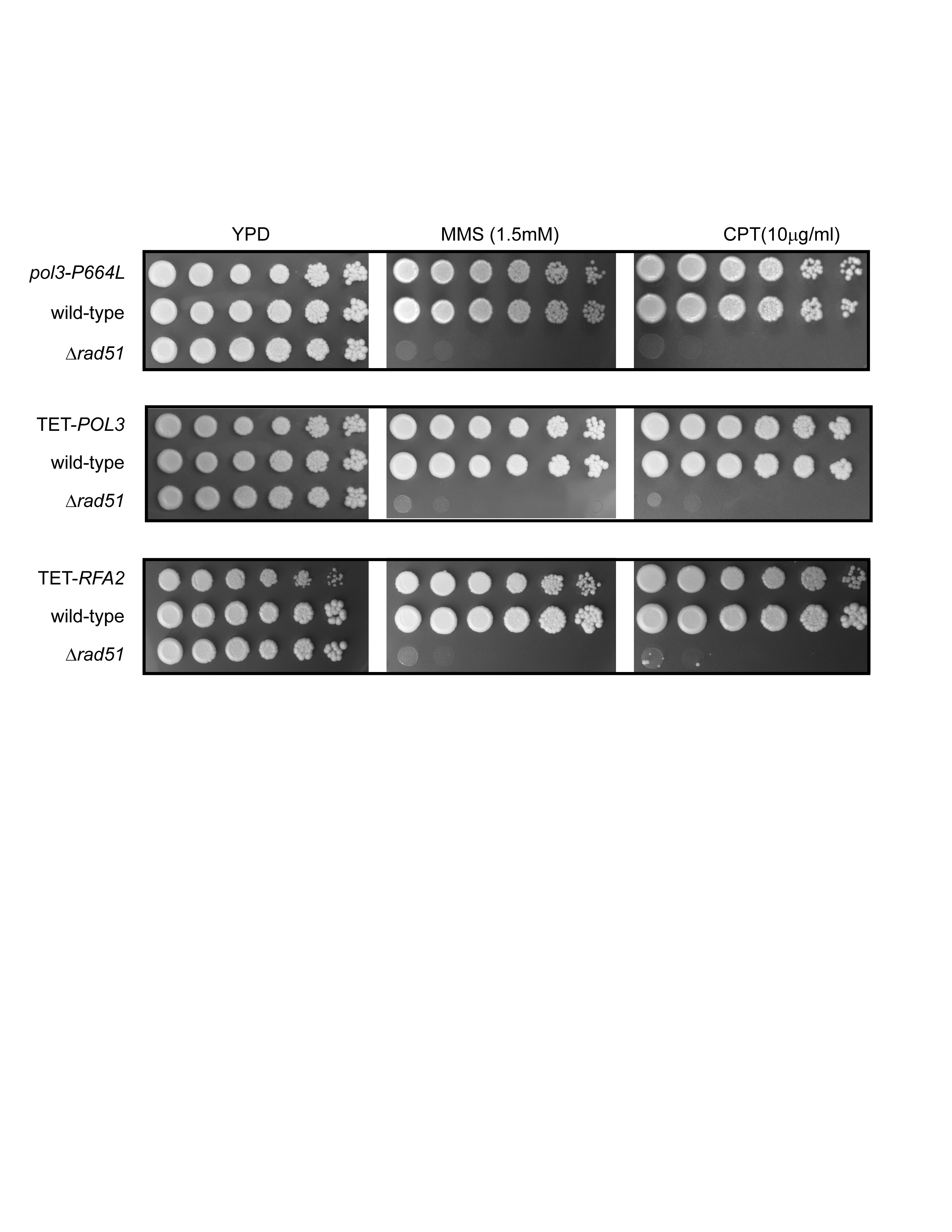

Supplement: Figure S1 — Sensitivities of the replication-deficient strains to DNA-damaging agents. Four-fold serial dilutions of wild-type strains and the mutant alleles were plated on YPD and YPD containing 1.5 mM MMS and 10 µg/ml camptothecin (CPT). Δrad51 was used as a control since it exhibits extreme sensitivity to the drugs used. The middle and bottom panels depict strains grown in the presence of 2 µg/ml and 0.1 µg/ml doxycycline, respectively. (TIF) [file pgen.1003551.s001.tif]
