# Supplementary material for: Fragile DNA Motifs Trigger Mutagenesis at Distant Chromosomal Loci in Saccharomyces cerevisiae
Source: PLoS Genet. 2013 Jun 13;9(6):e1003551. doi: 10.1371/journal.pgen.1003551 (PMC3681665; doi:10.1371/journal.pgen.1003551)
Supplement: Table S1 — Sequences of mutations analyzed in CAN1 in wild-type strain containing inverted repeats. a Coordinates of the first nucleotide in the mutated sequence are indicated based on the CAN1 coding strand sequence. b sub - base substitutions, indel - insertions or deletions (DOC) [file pgen.1003551.s002.doc]

Table S1. Sequences of mutations analyzed in *CAN1* in wild-type strain containing inverted repeats

|  | | | | | | |
| --- | --- | --- | --- | --- | --- | --- |
| Isolate | Coordinate in *CAN1* (coding strand) | Wild type base | Mutant base | Insertion/deletion (±#bases) | Wild type sequence context | Type of mutation |
| 1 | 284 | t | g |  | CATATTGGTAtGATTGCCCTT | sub |
| 2 | 290 | c | a |  | GGTATGATTGcCCTTGGTGGT | sub |
| 3 | 356 | c | g |  | AACGCCGGCCcAGTGGGCGCT | sub |
| 4 | 449 | c | a |  | ATCCCTGTTAcATCCTCTTTC | sub |
| 5 | 470 | c | a |  | ACAGTTTTCTcACAAAGATTC | sub |
| 6 | 541 | t | g |  | GGCAATCACTtTTGCCCTGGA | sub |
| 7 | 571 | g | a |  | AGTTGGCCAAgTCATTCAATT | sub |
| 8 | 580 | - | t | +1 | AGTCATTCAA-TTTTGGACGT | indel |
| 9 | 663 | - | a | +1 | CCCTGTCAAA-TATTACGGTG | indel |
| 10 | 670 | g | c |  | CAAATATTACgGTGAATTCGA | sub |
| 11 | 670 | g | t |  | CAAATATTACgGTGAATTCGA | sub |
| 12 | 789 | g | a |  | TCCGTTATTGgAGAAACCCAG | sub |
| 13 | 804 | c | - | -1 | ACCCAGGTGCcTGGGGTCCAG | indel |
| 14 | 807 | g | a |  | CAGGTGCCTGgGGTCCAGGTA | sub |
| 15 | 857 | g | a |  | TTCTTAGGTTgGGTTTCCTCT | sub |
| 16 | 887 | c | - | -1 | GCTGCCTTCAcATTTCAAGGT | indel |
| 17 | 895 | g | c |  | CACATTTCAAgGTACTGAACT | sub |
| 18 | 895 | g | a |  | CACATTTCAAgGTACTGAACT | sub |
| 19 | 910 | g | a |  | TGAACTAGTTgGTATCACTGC | sub |
| 20 | 937 | c | a |  | AGCTGCAAACcCAGAAAATCC | sub |
| 21 | 973 | g | t |  | CAAAAAAGTTgTTTTCCGTAT | sub |
| 22 | 1178 | a | t |  | GCAAATTCAAaTATTTACGTT | sub |
| 23 | 1214 | c | g |  | TTTGGTCTATcAAAGAACAAG | sub |
| 24 | 1253 | c | t |  | TCAAGGACCAcCAAAGGTGGT | sub |
| 25 | 1314 | c | a |  | CTTTGGCTTAcATGGAGACAT | sub |
| 26 | 1392 | g | a |  | TTTTTGCATGgTTATTTATCT | sub |
| 27 | 1623 | g | a |  | TAGCTGTTTGgATCTTATTTC | sub |

a Coordinates of the first nucleotide in the mutated sequence are indicated based on the *CAN1* coding strand sequence.

b sub - base substitutions, indel - insertions or deletions
