# Supplementary material for: Fragile DNA Motifs Trigger Mutagenesis at Distant Chromosomal Loci in Saccharomyces cerevisiae
Source: PLoS Genet. 2013 Jun 13;9(6):e1003551. doi: 10.1371/journal.pgen.1003551 (PMC3681665; doi:10.1371/journal.pgen.1003551)
Supplement: Table S2 — Sequences of mutations analyzed in CAN1 in pol3-P664L mutant strain carrying inverted repeats. a Coordinates of the first nucleotide in the mutated sequence are indicated based on the CAN1 coding strand sequence. b sub - base substitutions, indel - insertions or deletions, complex - complex mutations, slippage- slippage events between short direct repeats that are indicated by underlined sequences. (DOC) [file pgen.1003551.s003.doc]

Table S2. Sequences of mutations analyzed in *CAN1* in *pol3-P664L* mutant strain carrying inverted repeats.

| Isolate | Coordinate in *CAN1* (coding strand)a | Wild-type base | Mutant base | Insertion/ deletion (±#bases) | Wild-type sequence context | Type of mutationb |
| --- | --- | --- | --- | --- | --- | --- |
| 1 | 527 | c | t |  | TATTGGTTTTcTTGGGCAATC | sub |
| 2 | 530 | g | a |  | TGGTTTTCTTgGGCAATCACT | sub |
| 3 | 581 | tttgg | - | -5 | GTCATTCAATtttggACGTACAAAG | indel |
| 4 | 625 | t | - | -1 | TAGTATTTTTtGGGTAATTAT | indel |
| 5 | 629 | ta | -- | -2 | ATTTTTTGGGtaATTATCACAA | indel |
| 6 | 655 | c | a |  | GAACTTGTTCcCTGTCAAATA | sub |
| 7 | 679 | g | a |  | CGGTGAATTCgAGTTCTGGGT | sub |
| 8 | 686 | gGGTCGCt | aGGTCGCa |  | TTCGAGTTCTgGGTCGCtTCCATCAAAG | complex |
| 9 | 770 | c | - | -1 | GTTACCGGCCcAGTTGGATTC | indel |
| 10 | 803 | cc | -a |  | AACCCAGGTGccTGGGGTCCAG | complex |
| 11 | 892 | c | a |  | CTTCACATTTcAAGGTACTGA | sub |
| 12 | 892 | c | a |  | CTTCACATTTcAAGGTACTGA | sub |
| 13 | 920 | c | a |  | GGTATCACTGcTGGTGAAGCT | sub |
| 14 | 926 | a | t |  | ACTGCTGGTGaAGCTGCAAAC | sub |
| 15 | 937 | cCc | aC- |  | AGCTGCAAACcCcAGAAAATCCG | complex |
| 16 | 977 | t | - | -1 | AAAGTTGTTTtCCGTATCTTA | indel |
| 17 | 979 | c | g |  | AGTTGTTTTCcGTATCTTAAC | sub |
| 18 | 979 | c | a |  | AGTTGTTTTCcGTATCTTAAC | sub |
| 19 | 1001 | gct | c-- |  | TTCTACATTGgctCTCTATTATT | complex |
| 20 | 1002 | ctctctattattcattgg | - | -19 | TCTACATTGGctctctattattcattggACTTTTAGTTC | slippage |
| 21 | 1129 | at | -- | -2 | TTTGCCACATatCTTCAACGCT | indel |
| 22 | 1214 | -AAAgAAC | tAAAaAAC |  | TTGGTCTATCAAAaAACAAGTTGGCTC | complex |
| 23 | 1267 | cc | aa |  | AGGTGGTGTTccATACATTGCA | complex |
| 24 | 1268 | c | a |  | GGTGGTGTTCcATACATTGCA | sub |
| 25 | 1272 | c | g |  | GTGTTCCATAcATTGCAGTTT | sub |
| 26 | 1324 | tctactggtggtgaca | - | -15 | CATGGAGACAtctactggtggtgacaAAGTTTTCGA | slippage |
| 27 | 1351 | tg | -- | -2 | AGTTTTCGAAtgGCTATTAAAT | indel |
| 28 | 1351 | tg | -- | -2 | AGTTTTCGAAtgGCTATTAAAT | indel |
| 29 | 1386 | t | - | -1 | CAGGCTTTTTtGCATGGTTAT | indel |
| 30 | 1429 | g | c |  | ATTTATGCAAgCTTTGAAATA | sub |
| 31 | 1537 | c | a |  | TATCATTATTcAAGGTTTCAC | sub |

a Coordinates of the first nucleotide in the mutated sequence are indicated based on the *CAN1* coding strand sequence.

b sub - base substitutions, indel - insertions or deletions, complex - complex mutations, slippage- slippage events between short direct repeats that are indicated by underlined sequences.
