# Supplementary material for: Fragile DNA Motifs Trigger Mutagenesis at Distant Chromosomal Loci in Saccharomyces cerevisiae
Source: PLoS Genet. 2013 Jun 13;9(6):e1003551. doi: 10.1371/journal.pgen.1003551 (PMC3681665; doi:10.1371/journal.pgen.1003551)
Supplement: Table S3 — Sequences of mutations analyzed in CAN1 in pol3-P664L Δrev3 mutant strain carrying inverted repeats. a Coordinates of the first nucleotide in the mutated sequence are indicated based on the CAN1 coding strand sequence. b sub - base substitutions, indel - insertions or deletions, complex - complex mutations, slippage- slippage events between short direct repeats that are indicated by underlined sequences. (DOC) [file pgen.1003551.s004.doc]

Table S3. Sequences of mutations analyzed in *CAN1* in *pol3-P664L* ∆*rev3* mutant strain carrying inverted repeats

| Isolate | Coordinate in *CAN1* (coding strand)a | Wild-type base | Mutant base | Insertion/ deletion (±#bases) | Wild-type sequence context | Type of mutationb |
| --- | --- | --- | --- | --- | --- | --- |
| 1 | 274 | c | g |  | TAAGCAAAGAcATATTGGTAT | sub |
| 2 | 276 | - | tattggtatgattgcccttggtggtac | +27 | AGCAAAGACATATTGGTATGATTGCCCTTGGTGGTACTATTGGTACA | slippage |
| 3 | 284 | tgattgcccttggtggtactattggta | - | -27 | CATATTGGTAtgattgcccttggtggtactattggtaCAGGTCTTTT | slippage |
| 4 | 284 | tgattgcccttggtggtactattggta | - | -27 | CATATTGGTAtgattgcccttggtggtactattggtaCAGGTCTTTT | slippage |
| 5 | 299 | g | a |  | GCCCTTGGTGgTACTATTGGT | sub |
| 6 | 311 | c | a |  | ACTATTGGTAcAGGTCTTTTC | sub |
| 7 | 374 | c | a |  | GCTCTTATATcATATTTATTT | sub |
| 8 | 432 | tacattcatccctgt | - | -15 | GTGAAATGGCtacattcatccctgtTACATCCTCT | slippage |
| 9 | 530 | g | a |  | TGGTTTTCTTgGGCAATCACT | sub |
| 10 | 669 | c | a |  | TCAAATATTAcGGTGAATTCG | sub |
| 11 | 687 | g | a |  | TCGAGTTCTGgGTCGCTTCCA | sub |
| 12 | 724 | ctaatatactgttt | - | -14 | TATCGGGTTTctaatatactgtttTTGTATGGTT | slippage |
| 13 | 754 | gctggggt | - | -8 | GGTTTGTGGTgctggggtTACCGGCCCA | slippage |
| 14 | 836 | a | - | -1 | AAGGATAAAAaCGAAGGGAGG | indel |
| 15 | 980 | g | a |  | GTTGTTTTCCgTATCTTAACC | sub |
| 16 | 1002 | ctctctattattcattgg | - | -19 | TCTACATTGGctctctattattcattggACTTTTAGTTC | slippage |
| 17 | 1068 | c | g |  | CTACTTCCTAcGTTTCTACTT | sub |
| 18 | 1069 | gtttctac | - | -8 | TACTTCCTACgtttctacTTCTCCCTTT | slippage |
| 19 | 1195 | cgtattttatttggtctatcaaagaacaagttggctcc | - | -38 | CGTTGGTTCCcgtattttatttggtctatcaaagaacaagttggctccTAAATTCCTGT | slippage |
| 20 | 1195 | cgtattttatttggtctatcaaagaacaagttggctcc | - | -38 | CGTTGGTTCCcgtattttatttggtctatcaaagaacaagttggctccTAAATTCCTGT | slippage |
| 21 | 1324 | tctactggtggtgaca | - | -16 | CATGGAGACAtctactggtggtgacaAAGTTTTCGA | slippage |
| 22 | 1324 | tctactggtggtgaca | - | -16 | CATGGAGACAtctactggtggtgacaAAGTTTTCGA | slippage |

a Coordinates of the first nucleotide in the mutated sequence are indicated based on the *CAN1* coding strand sequence.

b sub - base substitutions, indel - insertions or deletions, complex - complex mutations, slippage- slippage events between short direct repeats that are indicated by underlined sequences.
