# Supplementary material for: Fragile DNA Motifs Trigger Mutagenesis at Distant Chromosomal Loci in Saccharomyces cerevisiae
Source: PLoS Genet. 2013 Jun 13;9(6):e1003551. doi: 10.1371/journal.pgen.1003551 (PMC3681665; doi:10.1371/journal.pgen.1003551)
Supplement: Table S4 — Sequences of mutations analyzed in CAN1 in pol3-P664L mutant strain carrying no inverted repeats. a Coordinates of the first nucleotide in the mutated sequence are indicated based on the CAN1 coding strand sequence. b sub - base substitutions, indel - insertions or deletions, complex - complex mutations, slippage- slippage events between short direct repeats that are indicated by underlined sequences. (DOC) [file pgen.1003551.s005.doc]

Table S4. Sequences of mutations analyzed in *CAN1* in *pol3-P664L* mutant strain carrying no inverted repeats

| Isolate | Coordinate in *CAN1* (coding strand)a | Wild-type  base | Mutant base | Insertion/ deletion (±#bases) | Wild-type sequence context | Type of mutationb |
| --- | --- | --- | --- | --- | --- | --- |
| 1 | 238 | c | t |  | AGGAGAAGTAcAGAACGCTGA | sub |
| 2 | 284 | tgattgcccttggtggtactattggta | - | -27 | CATATTGGTAtgattgcccttggtggtactattggtaCAGGTCTTTT | slippage |
| 3 | 284 | tgattgcccttggtggtactattggta | - | -27 | CATATTGGTAtgattgcccttggtggtactattggtaCAGGTCTTTT | slippage |
| 4 | 352 | g | c |  | GACCAACGCCgGCCCAGTGGG | sub |
| 5 | 395 | t | a |  | ATGGGTTCTTtGGCATATTCT | sub |
| 6 | 434 | c | a |  | GAAATGGCTAcATTCATCCCT | sub |
| 7 | 447 | t | c |  | TCATCCCTGTtACATCCTCTT | sub |
| 8 | 502 | g | - | -1 | ATTTGGTGCGgCCAATGGTTA | indel |
| 9 | 658 | g | c |  | CTTGTTCCCTgTCAAATATTA | sub |
| 10 | 668 | a | g |  | GTCAAATATTaCGGTGAATTC | sub |
| 11 | 673 | g | t |  | ATATTACGGTgAATTCGAGTT | sub |
| 12 | 687 | g | a |  | TCGAGTTCTGgGTCGCTTCCA | sub |
| 13 | 804 | ctggggtc | -tggggtt |  | ACCCAGGTGCctggggtcCAGGTATAAT | complex |
| 14 | 804 | c | - | -1 | ACCCAGGTGCcTGGGGTCCAG | indel |
| 15 | 922 | g | a |  | TATCACTGCTgGTGAAGCTGC | sub |
| 16 | 974 | tt | ga |  | AAAAAAGTTGttTTCCGTATCT | complex |
| 17 | 979 | c | - | -1 | AGTTGTTTTCcGTATCTTAAC | indel |
| 18 | 1002 | ctctctattattcattgg | - | -18 | TCTACATTGGctctctattattcattggACTTTTAGTTC | slippage |
| 19 | 1039 | gaccctaaactaacacaat | - | -19 | TCCATACAATgaccctaaactaacacaatCTACTTCCTAC | slippage |
| 20 | 1054 | c | t |  | TAAACTAACAcAATCTACTTC | sub |
| 21 | 1099 | g | t |  | TATTGCTATTgAGAACTCTGG | sub |
| 22 | 1195 | cgtattttatttggtctatcaaagaacaagttggctcc | - | -38 | CGTTGGTTCCcgtattttatttggtctatcaaagaacaagttggctccTAAATTCCTGT | slippage |
| 23 | 1195 | c | t |  | CGTTGGTTCCcGTATTTTATT | sub |
| 24 | 1195 | cgtattttatttggtctatcaaagaacaagttggctcc | - | -38 | CGTTGGTTCCcgtattttatttggtctatcaaagaacaagttggctccTAAATTCCTGT | slippage |
| 25 | 1403 | c | g |  | TTATTTATCTcAATCTCGCAC | sub |
| 26 | 1473 | a | - | -1 | TACCATTTAAaGCTAAATTAA | indel |
| 27 | 1536 | tc | -t |  | TTATCATTATtcAAGGTTTCAC | complex |
| 28 | 1625 | tcttatttcaatgcatattcagatgcagatttatttgga | - | -39 | GCTGTTTGGAtcttatttcaatgcatattcagatgcagatttatttggaAGATTGGAGA | slippage |

a Coordinates of the first nucleotide in the mutated sequence are indicated based on the *CAN1* coding strand sequence.

b sub - base substitutions, indel - insertions or deletions, complex - complex mutations, slippage- slippage events between short direct repeats that are indicated by underlined sequences.
