# Supplementary material for: Fragile DNA Motifs Trigger Mutagenesis at Distant Chromosomal Loci in Saccharomyces cerevisiae
Source: PLoS Genet. 2013 Jun 13;9(6):e1003551. doi: 10.1371/journal.pgen.1003551 (PMC3681665; doi:10.1371/journal.pgen.1003551)
Supplement: Table S5 — Sequences of mutations analyzed in CAN1 in wild-type strain carrying IS50-perfect palindrome. a Coordinates of the first nucleotide in the mutated sequence are indicated based on the CAN1 coding strand sequence. b sub - base substitutions, indel - insertions or deletions, complex - complex mutations. (DOC) [file pgen.1003551.s006.doc]

Table S5. Sequences of mutations analyzed in *CAN1* in wild-type strain carrying *IS50*-perfect palindrome

| Isolate | Coordinate in *CAN1* (coding strand)a | Wild-type  base | Mutant base | Insertion/ deletion (±#bases) | Wild-type sequence context | Type of mutationb |
| --- | --- | --- | --- | --- | --- | --- |
| 1 | 48 | c | g |  | AGCATATGTAcAATGAGCCGG | sub |
| 2 | 112 | c | a |  | AGACGTGGGTcAATACCATTG | sub |
| 3 | 146 | t | a |  | AGTAAAGAATtGTATCCATTG | sub |
| 4 | 244 | g | t |  | AGTACAGAACgCTGAAGTGAA | sub |
| 5 | 275 | a | g |  | AAGCAAAGACaTATTGGTATG | sub |
| 6 | 317 | c | - |  | TGGTACAGGTcTTTTCATTGG | indel |
| 7 | 351 | g | a |  | GACCAACGCCgGCCCAGTGGG | sub |
| 8 | 446 | ctg | -tt | -1 | ACATTCATCCctgTTACATCCTC | complex |
| 9 | 464 | t | g |  | TCTTTCACAGtTTTCTCACAA | sub |
| 10 | 522 | g | a |  | ACATGTATTGgTTTTCTTGGG | sub |
| 11 | 591 | c | a |  | TTTGGACGTAcAAAGTTCCAC | sub |
| 12 | 890 | - | t |  | GCCTTCACAT-TTCAAGGTAC | indel |
| 13 | 896 | g | a |  | ACATTTCAAGgTACTGAACTA | sub |
| 14 | 901 | g | c |  | TCAAGGTACTgAACTAGTTGG | sub |
| 15 | 969 | a | g |  | CCATCAAAAAaGTTGTTTTCC | sub |
| 16 | 1065 | - | c | 1 | AATCTACTTC-CTACGTTTCT | indel |
| 17 | 1123 | - | c | 1 | AAAGGTTTTG-CCACATATCT | indel |
| 18 | 1163 | c | a |  | ACCATTATTTcTGCCGCAAAT | sub |
| 19 | 1195 | cg- | aGt | 1 | CGTTGGTTCCcgTATTTTATTT | complex |
| 20 | 1314 | c | g |  | CTTTGGCTTAcATGGAGACAT | sub |
| 21 | 1314 | c | g |  | CTTTGGCTTAcATGGAGACAT | sub |
| 22 | 1394 | t | a |  | CATGGAGACAtCTACTGGTGG | sub |
| 23 | 1600 | a | g |  | CTATATCTCTaTTTTCCTGTT | sub |
| 24 | 1754 | t | g |  | TGGGACAAATtTTGGAATGTT | sub |
| 25 | 1796 | aTCACATTt | cTCACATTc |  | ATTACCTTTGaTCACATTtCCACGCCATT | complex |

a Coordinates of the first nucleotide in the mutated sequence are indicated based on the *CAN1* coding strand sequence.

b sub - base substitutions, indel - insertions or deletions, complex - complex mutations.
