# Supplementary material for: Fragile DNA Motifs Trigger Mutagenesis at Distant Chromosomal Loci in Saccharomyces cerevisiae
Source: PLoS Genet. 2013 Jun 13;9(6):e1003551. doi: 10.1371/journal.pgen.1003551 (PMC3681665; doi:10.1371/journal.pgen.1003551)
Supplement: Table S6 — Sequences of the primers used in this study. (DOC) [file pgen.1003551.s007.doc]

Table S6. Sequences of the primers used in this study

| Primer | Sequence | Purpose | |
| --- | --- | --- | --- |
| *POL3-*TET51 | CTCGTCATCGATCTTCACATCAACCATGGGAAGGGA | To replace the natural promoter of *POL3* with tetracycline downregulatable construct |  |
| *POL3-*TET31 | GACGGTTGATCTTGGTTTTGTGTGGTAAAAGTATGC |  |  |
| *POL3-*TET52 | GATTGCCGTTTGATTTTCTTTTCCAACTGGGGAGTA |  |  |
| *POL3-*TET32 | GTTCTCCTTTATATCTGCGTTTCTTTGCAGCGTTCT |  |  |
| *RFA2-*TET51 | GTTTCTTAGATAATTATCGCCATATACGAAACGCGTTAGGAAACGCGTTC CAGCTGAAGCTTCGTACGC | To replace the natural promoter of *RFA2* with tetracycline downregulatable construct |  |
| *RFA2-*TET31 | GCAGAAAAGAGCAAATCCTCCAATTCCTAGCACTAATACATACTTGCCATGCATAGGCCACTAGTGGAT |  |  |
| *RFA2-*TET52 | CCTCGATGAGCTTCCATTTTCAATTTTCATCTTTTTTTTCACGATGCGAAGTTTCTTAGATAATTATCGC |  |  |
| *RFA2-*TET32 | GTAATTTTAACATTCGCCAATAAAAATAAAAAAAATGATGCAGAAAAGAGCAAATCCTC |  |  |
| *URA3-*TD51 | TCTTCAAACACAGATGATTTCTTATTGAGATTTTTGAAAGATGACGGTGAAAACCTCTGAC | To introduce *URA3* 0.4 kb telomere distal from the repeats |  |
| *URA3-*TD31 | GAATTTTCGATATCTGGAATCTTACCTAATTGGACTGAACGCGGTATTTCACACCGCAGGG |  |  |
| *URA3-*TD52 | CCAGGTTACGTAACAGGTGCCTCTGCCAATGGTTCTTCAAACACAGATGATTT |  |  |
| *URA3-*TD32 | AACAACACGAGCAACATGATCTACTGGAACCATATTCACGGAATTTTCGATATCTGGAAT |  |  |
| *URA3-*TP51 | GTATACTATATGAATAAAGTGATCCCATAATCAGACTACCATGACGGTGAAAACCTCTGAC | To introduce *URA3* 0.6 kb telomere proximal from the repeats |  |
| *URA3*-TP31 | GCAATATATATTTAGTTCTAAAATGCGCTACTAAGTGCTGGCGGTATTTCACACCGCAGGG |  |  |
| *URA3-*TP52 | ATACTTTTTTGGTAGCTAAGTCAATGCCTCTCTGAATATTGTATACTATATGAATAAAG |  |  |
| *URA3-*TP32 | TGTTCCTTATTAAGAATTCTCAAGTTACAGTATTAAAAAGGCAATATATATTTAGTTC |  |  |
| *URA3-*8TD51 | ATTGGCCCCCTTTTATATTCATCGCGCTCTTTATCGCGGGATGACGGTGAAAACCTCTGAC | To introduce *URA3* 8 kb telomere distal from the repeats |  |
| *URA3-*8TD31 | TTATCACGCCAGGACTGCGGGAGTGGCGGGGGCAAACACAGCGGTATTTCACACCGCAGGG |  |  |
| *URA3*-8TD52 | AAGTTTGTATGGGAAGAACTCCAATATAACGGGACGTAACATTGGCCCCCTTTTATATTC |  |  |
| *URA3*-8TD32 | GAAAAAGCAAAATTTGGGCTCAGTAATGCCACTGCAGTGGCTTATCACGCCAGGACTG |  |  |
| *URA3*-30TP51 | GTATACTATATGAATAAAGTGATCCCATAATCAGACTACCATGACGGTGAAAACCTCTGAC | To introduce *URA3* 30 kb telomere proximal from the repeats |  |
| *URA3-*30TP31 | GCAATATATATTTAGTTCTAAAATGCGCTACTAAGTGCTGGCGGTATTTCACACCGCAGGG |  |  |
| *URA3-*30TP52 | ATACTTTTTTGGTAGCTAAGTCAATGCCTCTCTGAATATTGTATACTATATGAATAAAG |  |  |
| *URA3-*30TP32 | TGTTCCTTATTAAGAATTCTCAAGTTACAGTATTAAAAAGGCAATATATATTTAGTTC |  |  |
| *URA3*-30TD51 | TCCAAAGCAGTGACATTAAATTTTGCTTTCATTCGTGAAAATGACGGTGAAAACCTCTGAC | To introduce *URA3* 30 kb telomere distal from the repeats |  |
| *URA3-*30TD31 | ACTGAAGTTGAATTATAAATGATAATTTATTAGTCGATTCGCGGTATTTCACACCGCAGGG |  |  |
| *URA3-*30TD52 | GACATATTAGACATTAATAACTTCTCTGAATCGCTCTCACTCCAAAGCAGTGACATTAA |  |  |
| *URA3*-30TD32 | AAAAAGCTTGGAAAGAAAATTTGATGAGTTGTTTATTTCAACTGAAGTTGAATTATAAAT |  |  |
